# Catheter-induced Errors in Pressure Measurements in Vessels: an In-vitro and Numerical Study

**DOI:** 10.1109/TBME.2014.2308594

**Published:** 2014-06

**Authors:** Adelaide de Vecchi, Rachel E. Clough, Nicholas R. Gaddum, Marcel C.M. Rutten, Pablo Lamata, Tobias Schaeffter, David A. Nordsletten, Nicolas P. Smith

**Affiliations:** 1 King’s College London, Department of Biomedical Engineering and Imaging Sciences Division, St Thomas’ Hospital, London SE1 7EH, UK; 2 Eindhoven University of Technology, Department of Biomedical Engineering, PO Box 513, 5600 MB Eindhoven, The Netherlands

**Keywords:** Catheterization, medical signal detection, pressure measurement, in vitro, arterial blood pressure, computational fluid dynamics, pulse wave analysis

## Abstract

Accurate measurement of blood pressure is important because it is a biomarker for cardiovascular disease. Diagnostic catheterization is routinely used for pressure acquisition in vessels despite being subject to significant measurement errors. To investigate these errors, this study compares pressure measurement using two different techniques *in vitro* and numerical simulations. Pressure was acquired in a pulsatile flow phantom using a 6F fluid-filled catheter and a 0.014” pressure wire, which is considered the current gold standard. Numerical simulations of the experimental set-up with and without a catheter were also performed. Despite the low catheter-to-vessel radius ratio, the catheter traces showed a 24% peak systolic pressure overestimation compared to the wire. The numerical models replicated this difference and indicated the cause for overestimation was the increased flow resistance due to the presence of the catheter. Further, the higher frequency pressure oscillations observed in the wire and numerical data were absent in the catheter, resulting in an overestimation of the pulse wave velocity with the latter modality. These results show that catheter geometry produces significant measurement bias in both the peak pressure and the waveform shape even with radius ratios considered acceptable in clinical practice. The wire allows for more accurate pressure quantification, in agreement with the numerical model without a catheter.

## I Introduction

Accurate measurement of blood pressure in the cardiovascular system provides essential information to classify the severity of a variety of diseases. Despite recent advances in noninvasive techniques, catheterization remains the most common method for pressure acquisition; however, the measurement accuracy of fluid-filled catheters can be affected by technical limitations, including the reflection of the pressure wave at the tip and its distortion inside the probe [1], [2]. This latter effect is due to the column of fluid that fills the catheter, which is necessary to transmit the pressure to an external transducer [3], [4]: this design can give rise to inertial artifacts that alter the shape of the recorded waveform as it travels downstream inside the probe. The dynamic response of the catheter-transducer system required to reproduce the pressure waveform faithfully is still a matter of concern in clinical applications [5]. The catheter typically acts as a low-pass filter that attenuates all frequencies above the natural frequency. Further, as the signal frequency approaches the natural frequency, the system tends to resonate causing large errors. The catheter must therefore have a correct combination of length, diameter and compliance of the material to maximize the accuracy in the signal, as these parameters dictate the amount of damping of the system and its natural frequency. The properties and dimensions of catheters should be chosen to provide the highest possible natural frequency and thus maximize the flat frequency response necessary to produce a high-fidelity measurement [6]–[8]. In general, stiffer catheters increase the accuracy of the measurement; however, more compliant materials are necessary for better navigation in complex anatomies. Another source of disturbance impairing the dynamic response is the presence of air bubbles in the lumen, which can appear if the catheter is too compliant or too long, or with too small a diameter [5].

The signal distortion has significant potential to compromise important clinical markers that can be derived from waveform shape analysis, such as the pulse wave velocity (PWV) and the ratio of the stroke volume to the pulse pressure (defined as the difference between the peak systolic and diastolic pressure, *P_s_* – *P_d_*). The PWV is the speed at which a pressure wave travels along the artery and is measured by recording pressure transients at two different locations separated by a known distance Δ*x*: the transit time Δ*t* can then be obtained by aligning the foot of the waveforms (foot-to-foot methods), which is calculated based on the shape of the systolic upstroke [9]. The PWV, expressed as Δ*x*/Δ*t*, is directly related to the square root of the artery stiffness. These parameters have been shown to be independent predictors of adverse cardiovascular events in pathologies related to arterial stiffening and subsequent hypertensive pressures, increased ventricular afterload and higher myocardial oxygen demand [10]–[12]. Abnormal pulse pressure, waveform shape and PWV are also factors that can predispose patients with repaired aortic coarctation to increased cardiovascular risk [13].

Another potential source of measurement bias is related to a high value of the ratio between the catheter and the vessel radius (radius ratio), i.e. a relatively large catheter compared to the vessel size. This can generate partial obstruction of the lumen, resulting in pressure overestimation in relatively smaller vessels including coronary arteries, peripheral circulation and pediatric cases [14], [15]. Analytical flow models of a straight catheterized tube have shown that, for radius ratios ranging from 0.3 to 0.7, partial blockage can induce an increase in the flow resistance by a factor of 3-33 [16]. In the patient context, such artifacts can produce significant discrepancies in the disease evaluation based on catheter data. Pressure measurements are also used for risk stratifications in pulmonary hypertension patients waiting for heart transplant, where pulmonary vascular resistance, pulmonary artery systolic pressure and transpulmonary pressure gradient are key markers for pre-operative assessment [17], [18]. Errors in the measurement of these parameters have important diagnostic implications associated with both cost and treatment options.

Pressure wires can, to a significant extent, overcome these drawbacks: the transducer is placed directly at the tip and the wire thickness is negligible compared to that of a fluid-filled catheter. This leads to a significant reduction of both the inertial effects and the obstruction artifacts, making this technique the current gold standard for invasive measurements [19], [20]. However, despite these results, pressure wires remain less commonly applied clinically due to technical complications (i.e. kinking, entangling with other intravascular equipment), increased cost and a higher degree of operator training [21].

To understand and quantify the causes of measurement errors, this study compares the performance of a 0.014” pressure wire to that of a 6F fluid-filled catheter, which is routinely used in the clinic for aortic pressure measurement, during pressure acquisition in a pulsatile flow phantom. The *in vitro* set-up provides a range of physiological systolic pressures without adding confounding factors typical of the patient context, such as beat-to-beat variability and localized changes in wall stiffness. This allows a close control of the fluid-dynamic conditions, which can be accurately modeled using computer simulations to provide a physical interpretation of the observed discrepancies.

## II Material and methods

### A Experimental set-up

**Fig. 1:**
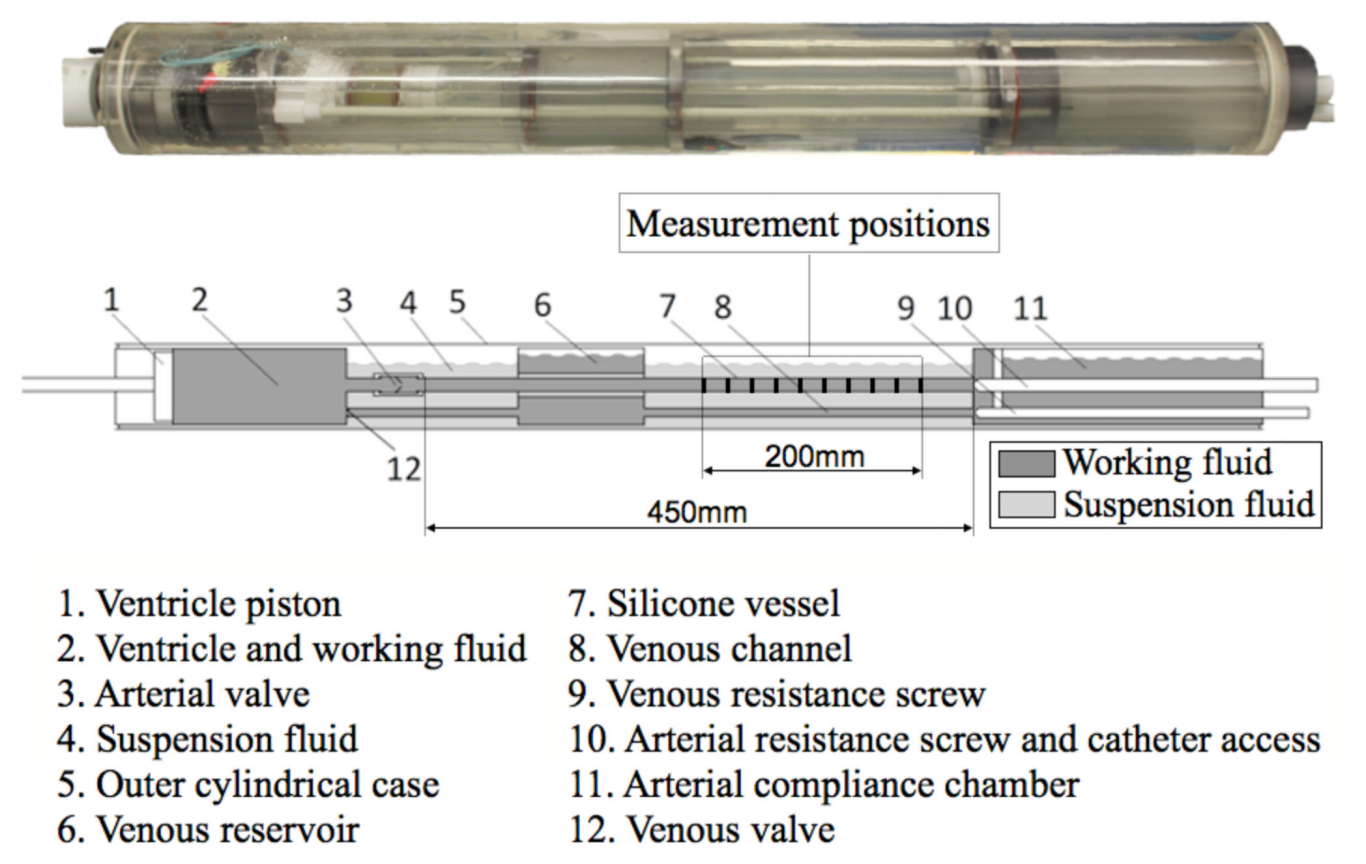
Diagram of the pulsatile flow phantom.

The pulsatile flow phantom was built to simulate a working ventricle with simplified pulmonary and systemic vessels. The different parts forming the experimental rig are shown in Fig. 1. The piston pump (1) ejected the fluid contained in a cylindrical chamber (2) through a tri-leaflet polyurethane valve (3) into a straight silicone tube (7). At the opposite end, a Windkessel system consisting of an adjustable resistance screw (10) and a compliance chamber (11) provided the desired afterload. The working fluid was then redirected to the ventricle via a venous channel (8) and reservoir (6). The systemic vessel (silicone “aorta”) had an inner radius *R_i_*=8mm and an outer radius *R_o_*=9.5mm. The pressure measurements were collected over 200mm towards the distal end. More details on the flow phantom setup can be found in [22].

A pulsatile flow with 1Hz frequency (corresponding to 60 beats per minute) was supplied by the pump using water at room temperature with viscosity *μ*=0.001002 Pa·s and density *ρ_f_*=998 kg/m^3^, respectively. Pulsatile flows can be defined by the Reynolds and the Womersley number, two dimensionless parameters that express the ratio of inertial to viscous force and the pulse frequency in relation to viscous effects, respectively:

(1) $$Re=\frac{2R_{i}U\rho_{f}}{\mu };\;\alpha =R_{i}={\left(\frac{2\pi f\rho_{f}}{\mu }\right)}^{\frac{1}{2}}$$

In this experiment, the Reynolds number based on the mean flow velocity *U* was 1308 and the Womersley number 20, hence in the physiological range for a normal aorta [23], [24]. The flow phantom was designed to be compatible with Magnetic Resonance Imaging (MRI). In addition to direct pressure measurements, phase-contrast MRI (PC-MRI) data were acquired in the same catheter configuration of the numerical model in a 3T MR Scanner (Achieva, Philips Medical Systems, Best, The Netherlands) using a flow-sensitive gradient echo sequence (field of view: 120x120x64 mm^3^; voxel size: 0.94x0.94x8 mm^3^; flip angle: 10°; velocity encoding: 60 cm/s; ratio of repetition time to echo time, TR/TE: 3.59/2.74 ms).

### B Direct pressure measurement in vitro

Pressure data was acquired using a 6F Swan Ganz catheter (Boston Scientific, Natick, MA, USA) with two lumens, a total length *l* of 800mm and an external radius *R_c_* of 1mm. Since the shape of the fluid-filled lumen was not circular, an internal radius *R* of 0.32 mm was calculated based on the estimated internal area. A Young’s modulus, *E_cath_*, of 32Mpa was obtained from torsional and flexural tests carried out at our institution on an MR-compatible catheter similar to the one used in the experiment [25]. The undamped natural frequency *f_n_* and the damping coefficient *ζ* can be derived from the equations describing the behavior of a mass-spring system and are expressed as:

(2) $$f_{n}=\frac{1}{2\pi }\sqrt{\frac{\pi R^{2}}{\rho l}\frac{dP}{dV}};\;\zeta =\frac{4\mu l}{R^{3}\sqrt{\varrho l\pi \frac{dP}{dV}}}$$

where μ and ρ are the viscosity and density of the fluid that fills the lumen, respectively. The inverse of the compliance, *dP*/*dV*, can be related to the Young’s modulus by the following equation [26]:

(3) $$E_{cath}=\frac{dP}{dV}\frac{2}{t}\sqrt{\frac{V^{3}}{\pi l}}$$

where *P* is the pressure necessary to displace a volume of fluid *V* and *t* is the thickness of the catheter annulus enclosing the lumen. The catheter used in the experiment had a natural frequency of 40Hz and a damping coefficient of 0.15.

The catheter was inserted in the tube facing the flow direction, resulting in a radius ratio (*R_c_*/*R_i_*) of 0.125. It was then progressively pulled back to gauge the pressure at 10 axial positions with 20mm intervals. Additional recordings were subsequently performed at the same locations using a 0.014” PressureWire Certus (St. Jude Medical, St. Paul, MN, USA), which corresponds to a radius of 0.18mm and to a radius ratio of 0.0225. The analogue signals from the transducers were acquired at 100Hz via a data acquisition card (USB-6009, National Instruments, Austin, TX, USA). The pressure wire was interfaced to the acquisition card via a Radi Analyser Xpress (St. Jude Medical, St. Paul, MN, USA). Atmospheric pressure in the catheter was set to zero using in-house software and the calibration followed a standard procedure using a saline filled sheath. To avoid the appearance of air bubbles the device was carefully flushed. Klee et al [27] presented a method based on the mathematical concept of curvature of a signal (defined as the rate at which a curve recedes from its tangent) to detect the distortion of the pressure waveform due to air bubbles: a threshold value for the sum of the curvatures of 3.85 was found to separate severely distorted signals from controls. When this method was applied to our signals, the sum of the curvatures was between 6 and 8.7, hence above of this threshold value. The signals were therefore considered to be free from air bubbles.

### C Numerical models

**Fig. 2:**
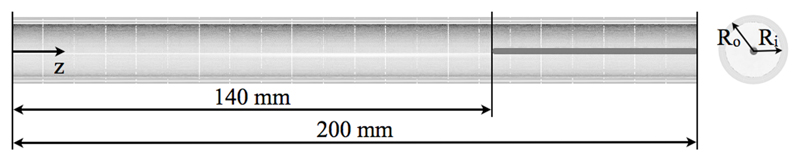
Catheter position in the numerical model. The flow is aligned with the positive z axis.

**Fig. 3:**
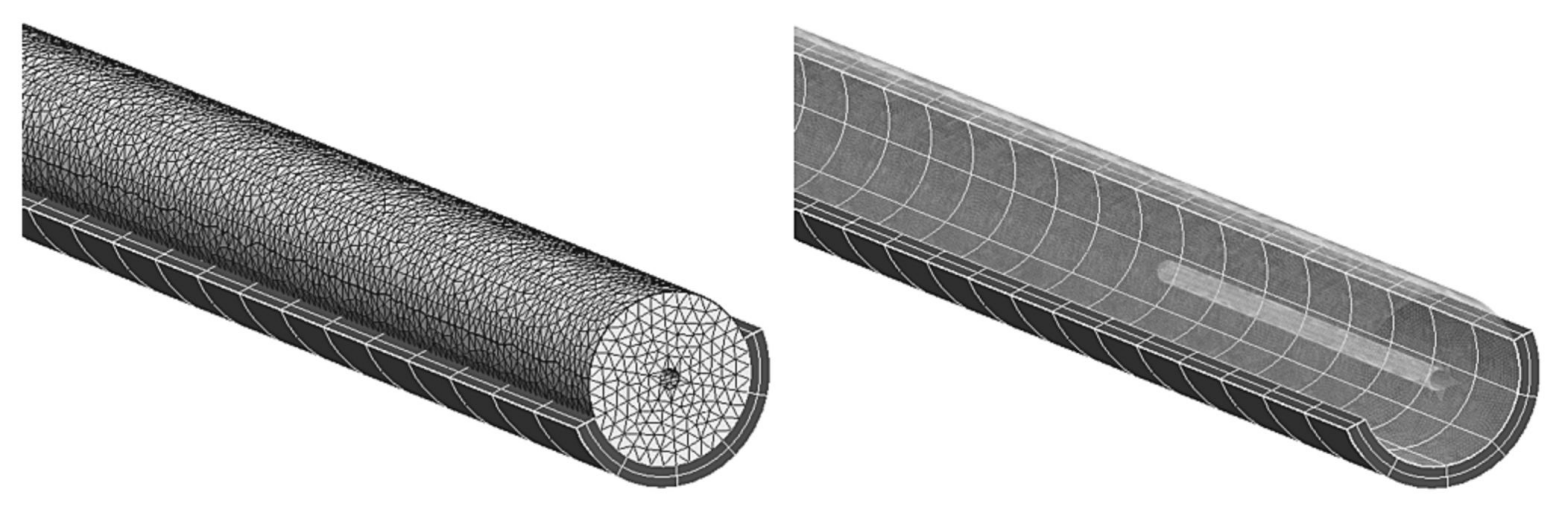
Tetrahedral mesh (fluid domain) and hexahedral mesh (solid domain) in the numerical FSI model with a catheter.

Two numerical models of the systemic vessel were generated to investigate the differences between the two sets of experimental measurements. The first one consisted of just the elastic vessel without any measuring probe inserted (catheter or wire). This configuration was compared to the vessel with the pressure wire inserted, as this device has a negligible radius ratio and thus causes minimal disturbance in the flow field. The second one included a coaxial rigid body with the same radius of the catheter and sought to reproduce the flow dynamics inside the catheterized vessel. This scenario replicated the *in vitro* configuration with the catheter tip at z=140mm, as shown in Fig. 2. The fluid-structure domain consisted of a tetrahedral mesh with approximately 128000 elements for the fluid, coupled to a hexahedral mesh with 440 elements for the solid (Fig. 3). In both cases, the silicone tube was modeled as an isotropic neo-Hookean material with Young’s modulus *E_s_*=384KPa and density *ρ_s_*=1250kg/m^3^.

### D Numerical study

The numerical study has been set up to match the experimental conditions as close as possible. Numerical simulations of fluid-structure interaction (FSI) were performed on both models using a the finite element software *CHeart*, which has been previously applied and validated in cases of non-linear FSI in physiological flows [28], [29]. The solver is based on a coupled fluid-solid algorithm: the solid mechanics is modeled using the quasi-static incompressible finite elasticity equations, while the Arbitrary Lagrangian-Eulerian (ALE) formulation of the full incompressible Navier-Stokes system is used to solve the fluid problem. These sets of equations, combined with the corresponding constraints imposed on the boundaries, are discretized and solved using a Galerkin technique. A boundary condition based on the pressure wire data was applied to the outlet of the model without a catheter. When the catheter was included, the outflow pressure boundary condition was based on the catheter traces in the outlet. This was dictated by the necessity to take into account the increased resistance due to the reduction of the area that occurs when the catheter is inserted. The velocity profile at the inlet of the model with a catheter was derived from the PC-MRI data acquired in a two-dimensional slice in the corresponding location. This same inflow boundary condition was used for the model without a catheter, since the inlet is sufficiently distant from the tip of the catheter to be considered unaffected by blockage effects. Three pulsatile cycles were simulated with a time step of 2ms, necessary to ensure the numerical stability of the model.

## III Results

### A Peak pressure overestimation

**Fig. 4:**
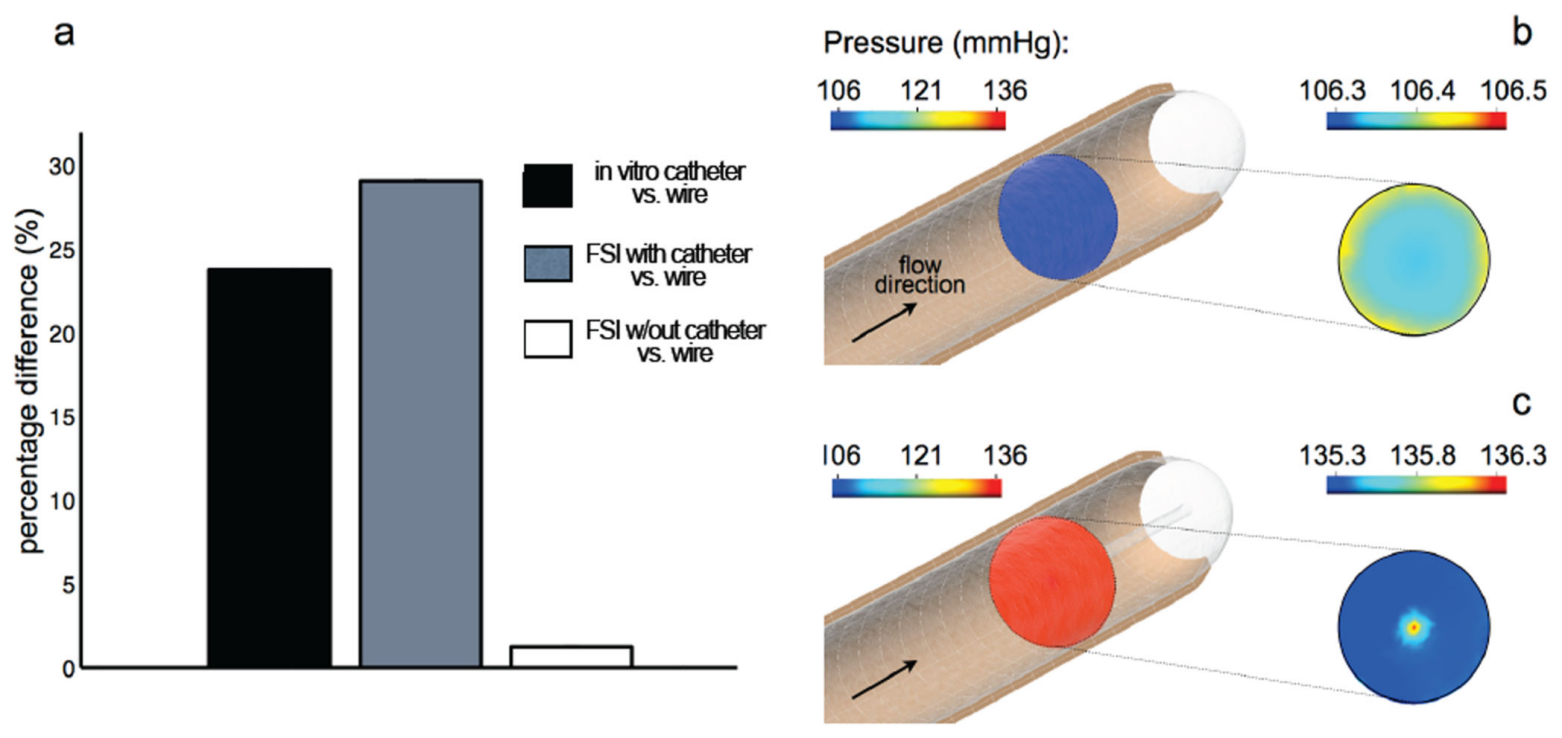
(a) Percentage difference in the measured peak systolic pressure between wire and catheter in vitro, and wire and FSI models. Pressure isocontrours in a cross-section of the FSI model without (b) and with a catheter (c).

The peak systolic pressures from the catheter and the two numerical models are compared to the wire data in the bar graph of Fig. 4a. The black bar on the left-hand side shows that a peak pressure overestimation of 24% is observed in the catheter data relative to the wire. This discrepancy is replicated by the catheterized FSI model, which predicted a peak pressure 29% larger than the corresponding wire measurement (Fig. 4a, grey bar). The FSI model without a catheter successfully reproduces the wire data, with a maximum discrepancy of only 1.5% (Fig. 4a, white bar). The FSI results also show that, in the absence of the catheter, the pressure in an axial cross-section perpendicular to the flow direction is approximately constant: in the cross-section highlighted in Fig. 4b the pressure at peak systole is 106.4 mmHg, with minor fluctuations of +/-0.1 mmHg. When the catheter is accounted for in the numerical model, the systolic pressure spatially averaged in a cross-section at the same location (now corresponding to the tip of the catheter) is 135.5 mmHg as shown in Fig. 4c. A region of higher pressure forms around the tip: however, the maximum pressure difference between the center and the periphery of the cross-section is not significant (1 mmHg), suggesting that this localized disturbance due to the impact of the flow against an “obstacle” is not the main factor in the peak pressure overestimation. The pulse pressure, *P_s_* – *P_d_*, at this location is 100.86 mmHg, 132.29 mmHg and 101.62 mmHg calculated from the wire, the catheter and the numerical results, respectively. The values from the wire and the numerical simulations are therefore approximately 76% of the corresponding catheter result.

### B Waveform shape analysis and pulse wave velocity

**Fig. 5:**
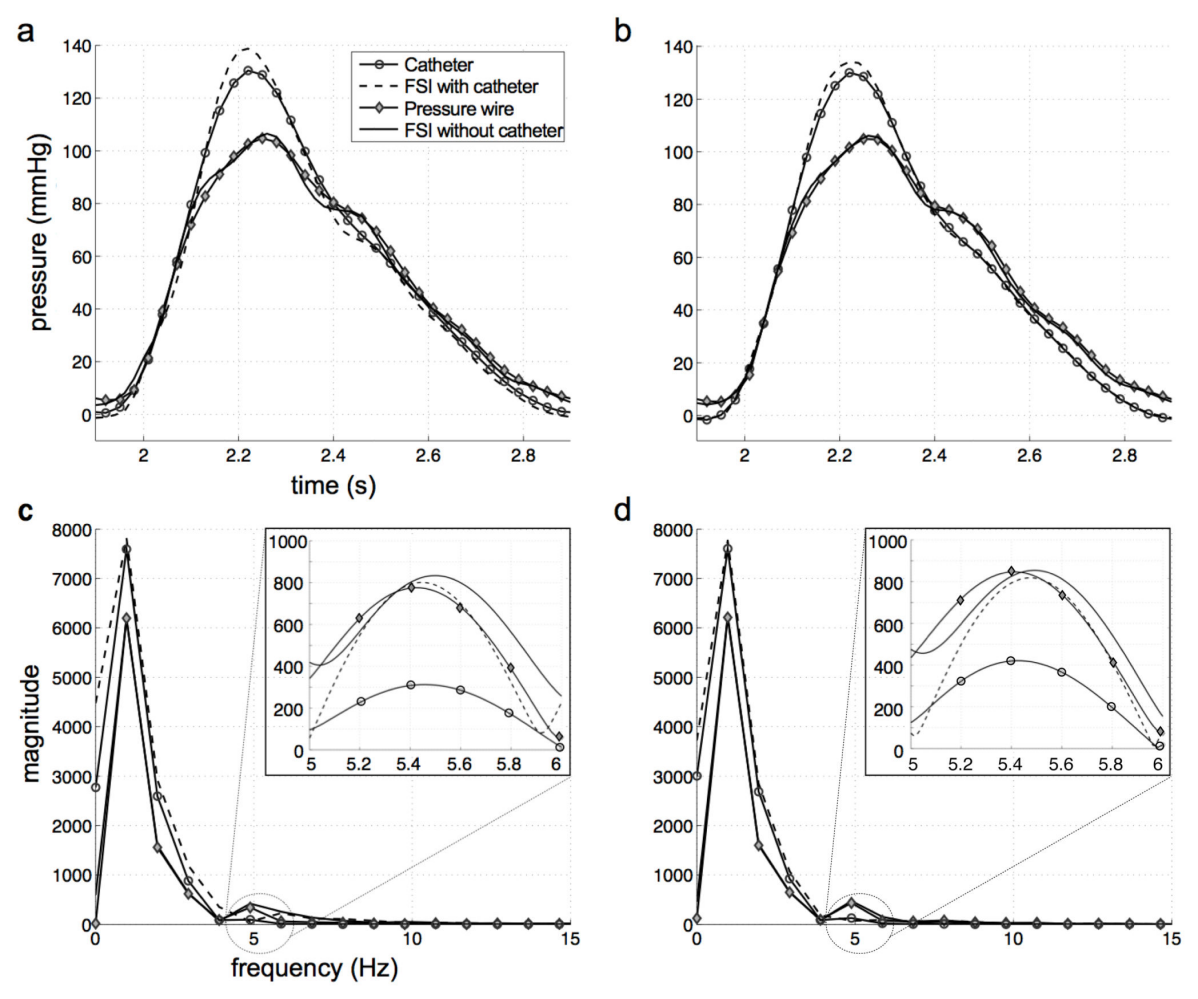
Pressure waveforms from catheter, wire and FSI simulations in two cross-sections at *z*=100mm (a) and *z* =140 mm (b). Single-sided amplitude spectrum of the frequencies in the pressure signals at *z* =100 mm (c) and *z* =140 mm (d).

The temporal transients of pressure from experimental measurements and numerical simulations in two cross-sections, at z=100mm and z=140mm respectively, are reported in Fig. 5a-b. The waveforms from the FSI model without a catheter and the wire are in agreement; similarly, the curves from the FSI model with a catheter replicate the catheter data collected *in vitro*. In both cases, the maximum discrepancy between the experimental measurements and the corresponding numerical models is below 5%.

**Fig. 6:**
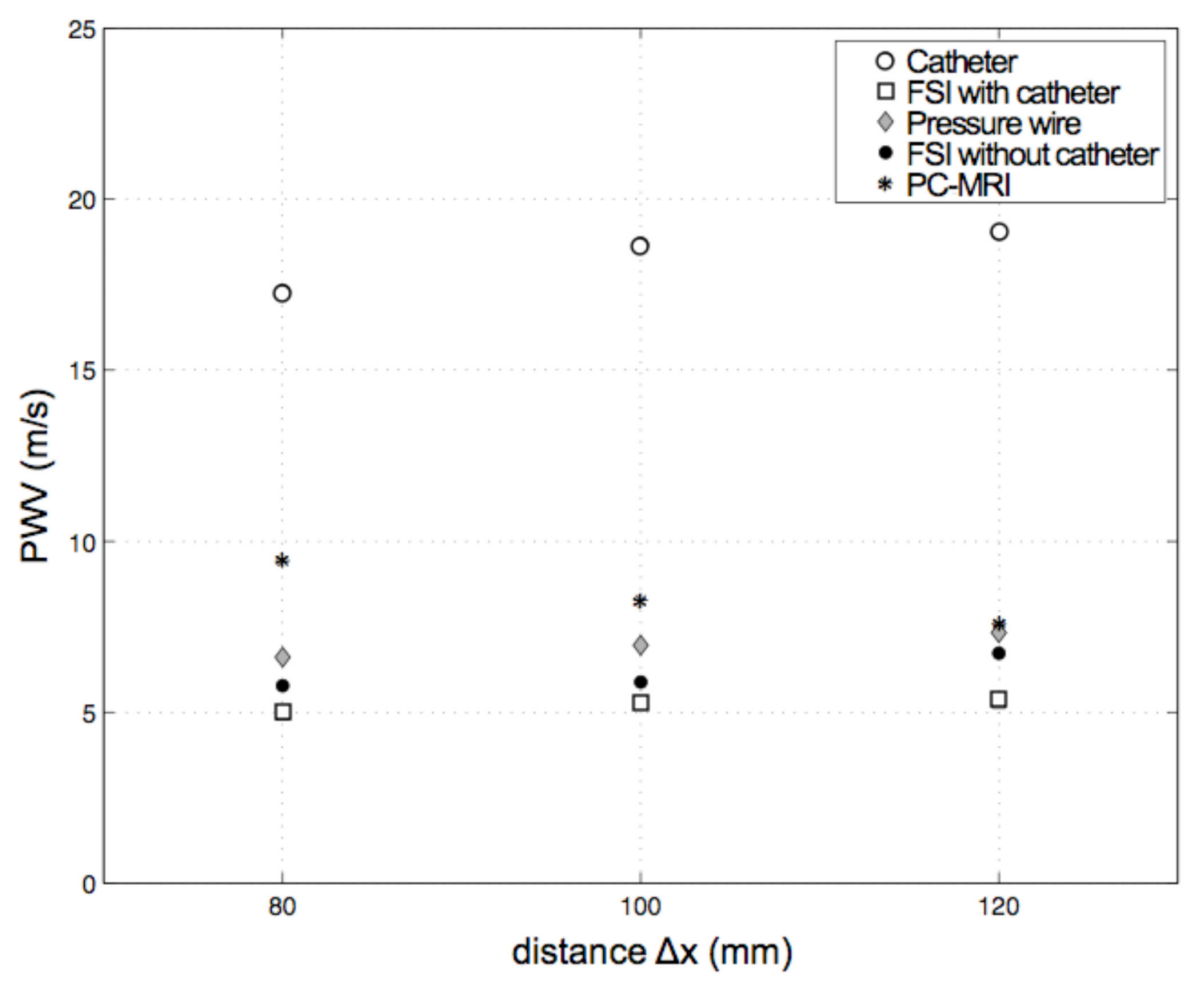
Pulse wave velocity from wire, catheter, PC-MRI and numerical signals over three distances *Δx*.

A Fast Fourier Transform (FFT) analysis of the pressure waveforms reveals a main frequency peak of 1 Hz, corresponding to the pulse value of 60 beats per minute (Fig. 5c-d). A second, higher frequency is also present in the signals. To increase the resolution of this frequency mode, the waveforms have been interpolated using a standard zero-padding technique: the plots with magnified axes show that the second peak corresponds to a frequency of 5.4 Hz and has similar magnitude in the data from the wire and both the numerical models, but is significantly damped in catheter measurements. This frequency value is compatible with the oscillation that gives rise to the dicrotic notch in the descending part of the pressure waveform observed in Fig. 5a-b. As a result of the higher frequencies damping, the dicrotic notch is absent in the signal recorded by the catheter. To better understand the consequences of this frequency damping, the pulse wave velocity is derived from the direct *in vitro* measurements (wire and catheter), from the PC-MRI data acquired in the scanner and, finally, from the numerical FSI simulations with and without a catheter (Fig. 6). An algorithm based on the foot-to-foot method [9] has been used to calculate the transit time of the waveform, Δ*t*, between two locations separated by a distance Δ*x* of 80mm, 100mm and 120mm. The resulting PWV for each axial length is the average of three values corresponding to different locations along the tube of the distance Δ*x* considered. The PWV calculated from the catheter data is approximately 3.5 times higher than that obtained from wire and PC-MRI data, and FSI simulations. On the longer distance of 120mm, where the relative error in the transit time calculation is lower, the FSI model without a catheter, the wire and the PC-MRI data indicate a range of PWV of 6.7 to 7.2; the PWV in the FSI model with a catheter is approximately 5.4, hence in approximate agreement with the values obtained from these modalities. However, this value is considerably lower than that of 19 from the *in vitro* catheter traces, despite the similar value of peak systolic pressure in the two cases (see Fig. 5a-b).

## IV Discussion

This study shows that: 1) catheter measurements can significantly overestimate peak pressure even with a moderate radius ratio of 0.125; 2) numerical results reproduced this pressure overestimation without the frequency damping of the catheter *in vitro*; 3) the suppression of higher frequencies of pressure in the catheter data results in a PWV significantly higher than that from the wire, PC-MRI and numerical data.

Fluid-dynamic principles provide physical explanations for the pressure overestimation in the catheter measurements. When the catheter is inserted, the same rate of fluid must flow through a narrower duct (annulus). The flow resistance downstream of the tip is thus higher than that of a noncatheterized vessel with the same radius. If this increased resistance is taken into account in the FSI model, the numerical results reproduce the pressure overestimation observed *in vitro*. Further, the model shows that the radial pressure change in the cross-section at the catheter tip is not significant (Fig. 4c). This suggests that, for this value of radius ratio, the major determinant of the pressure overestimation is the increased flow resistance in the annular region, rather than the artifacts around the tip. An increment in the mean flow resistance by a factor of 3 has been reported in analytical flow models with a radius ratio of 0.3 as a consequence of partial blockage [16]. The present experiment demonstrates that the measurement error is significant even with a lower radius ratio of 0.125, which is routinely used in the clinic. This is of particular significance in the evaluation of pulmonary hypertensive patients waiting for heart transplant based on pulmonary artery pressures and vascular resistance, as right heart catheterization typically results in radius ratios similar to that of the present study [17]. Elevated systolic pressures in the pulmonary artery are associated with high post-operative mortality in this cohort [18] and thus an error of over 20% can potentially bias the assessment of the disease severity.

Another consequence of the measurement inaccuracy using fluid-filled catheters that could influence clinical decisions comes from the slow dynamic response of this type of probe. As described above, the column of fluid in the catheter lumen dampens higher frequencies in the signal as it travels along the probe towards the external transducer: the system acts consequently as a low-pass filter. The catheter traces recorded *in vitro* showed a distinct absence of higher frequency modes, which were instead present in the wire and in both FSI models (Fig. 5c-d). This frequency damping clearly affects the shape of the measured pressure waveforms. Despite being qualitatively similar, the traces from the *in vitro* catheter and from the FSI model with a catheter result in a considerable discrepancy in the position of the foot of the waveform and hence in the pulse wave velocity. The main difference between the experimental catheter and the corresponding numerical model is the absence of the internal column of fluid in the latter, which is responsible for the attenuation of the higher frequencies of pressure. Consistently, this frequency damping is only observed in the *in vitro* catheter traces and is not replicated in the numerical model. The subsequent bias in the calculation of the PWV is not negligible and can therefore be a confounding factor for disease diagnosis in pathologies related to arterial stiffening. Similarly, the absence of the dicrotic notch in the catheter signal also has clinical relevance as this parameter is correlated with isolated systolic hypertension, which exposes patients to a stroke risk two to four times higher than in normotensive subjects [30]. It should however be noted that frequency damping is not the only factor that may affect the accuracy of the PWV computation. The impedance mismatch due to the insertion of the catheter and the increased downstream resistance also play an important role in the wave propagation by inducing a discontinuity of conditions. As mentioned previously, the presence of the catheter causes an obstruction leading to a decrease of the cross-sectional area. In PWV analysis this corresponds to a reflection point, which generates an additional reflected wave that is not present in the PC-MRI or wire data. This wave, as well as the transmitted wave, is reflected back and forth by the inlet and outlet boundary condition and interferes with the original waveform. These additional reflections can alter the shape of the measured signal and consequently the PWV, resulting in a less accurate estimate of its value. However, the numerical results from the model with a catheter suggest that the error in the PWV is mainly related to the frequency damping from the internal fluid rather than to the wave reflections.

The optimal level of damping is related to the shape of the waveform and to the heart rate, with higher rates and steeper upstrokes requiring higher natural frequencies and damping to avoid the risk of resonance during measurement. In clinical applications this level of damping is however rarely achieved due to constraints in the length and internal radius of the lumen. Underdamping in fluid-filled catheters can result in overestimation of systolic pressure [7]. High natural frequencies can nonetheless limit this artifact [3]: in a case presented by [5], the arterial pressure measured in a patient using an underdamped system with *f_n_=*15Hz and *ζ*=0.15 resulted in approximately an 8% overestimation of the systolic pressure compared to the high-fidelity signal measured by a catheter tipped pressure transducer. When the natural frequency was increased to 24Hz, however, the high-fidelity pressure waveform could be reproduced with minimal distortion. The catheter used in this experiment has the same damping coefficient but a higher natural frequency of 40Hz: it is thus reasonable to expect that the lumen size and the material properties of the catheter have a more limited influence on the observed pressure overestimation of 24%.

Finally, although the experimental and numerical simulations were set up to achieve realistic systolic pressure and waveform shape, some limitations should be mentioned. The diastolic pressure value is lower than the physiological range and the pulse pressure is consequently higher than normal. This is due to a technical constraint in the experimental set-up. As the systemic and venous vessel are parallel to each other, the pressure in the compliance chamber had to reduce the velocity of the flow to zero and then accelerate it in the opposite direction down the venous return. This posed a restriction on the minimum value of the impedance that could be achieved: the diastolic pressure had thus to be lowered to obtain a physiological flow profile. However, this scaling of the pressure waveform does not affect the PWV calculation, which is only based on the shape of the wave, nor the validity of the comparison between the measurement accuracy of the catheter and the wire in the same conditions. Further, the flow phantom did not include peripheral vessels and side branches, whose impedance is thus not accounted for. The use of a single straight tube instead of a more realistic aortic geometry is motivated by the necessity to avoid reproducing anatomical features that can introduce more complex fluid dynamics effects and thus hinder the identification of the causes of error in the measurements. A major difference between the simulations and the *in vitro* study is that the catheter in the FSI model is rigid and fixed coaxially to the aorta, while in the experiment it is flexible, free to fluctuate and therefore not concentric. In the controlled settings of the experiment, the oscillations experienced by the catheter were nonetheless small and the tip did not touch the wall during measurement. Generating a flexible, free to move and fluid-filled FSI model for the catheter was not thought necessary since the agreement between the pressures waveforms in the FSI simulations and in the *in vitro* experiment was strong. In this context, it should also be stressed that, although considered the gold standard for invasive measurement, the pressure wire is more flexible than the catheter and is thus prone to experience larger fluctuations that might affect the reproducibility of the measurements. As for the catheter, however, this is more likely to happen in complex anatomies and physiological conditions. In this *in vitro* study, pressure traces were recorded twice at each location and showed negligible variability.

In conclusion, the choice of the most suitable catheter should be based upon considerations of the radius ratio and the frequency response. Decreasing the diameter size to avoid excessive blockage of the vessel can affect the natural frequency of the system; similarly, the use of stiffer materials to achieve a high dynamic response can compromise the navigation properties and the conformability to complex anatomies. Pressure wires can minimize errors in peak systolic pressure and PWV, and provide more accurate measurement in small vasculature.

## References

[1] Kanai H, Iizuka M, Sakamoto K (1970). One of the problems in the measurement of blood pressure by catheter-insertion: wave reflection at the tip of the catheter. Med Biol Eng.

[2] Pijls N, Kern M (2000). Practice and Potential Pitfalls of Coronary Pressure Measurement. Catheter Cardiovasc Interv.

[3] Falsetti HL, Mates RE, Carroll RJ, Gupta RL, Bell AC (1974). Analysis and correction of pressure wave distortion in fluid-filled catheter systems. Circulation.

[4] Krovetz LJ, Jennings RB, Goldbloom SD (1974). Limitation of Correction of Frequency Dependent Artefact in Pressure Recordings Using Harmonic Analysis. Circulation.

[5] Gardner RM (1990). Direct arterial pressure monitoring. Curr Anaesth Crit Care.

[6] Li J, van Brummelen A, Noordergraaf A (1976). Fluid-filled blood pressure measurement systems. J Appl Physiol.

[7] Allan M, Gray W (1988). Measurement of arterial pressure using catheter-transducer systems. Br J Anaesth.

[8] Taylor B, Donovan F (1992). Hydraulic resistance and damping in catheter-transducer systems. IEEE Eng Med Biol.

[9] Gaddum NR, Alastruey J, Beerbaum P, Chowienczyk P, Schaeffter T (2013). A technical assessment of pulse wave velocity algorithms applied to non-invasive arterial waveforms. Ann Biomed Eng.

[10] Nichols W, Singh B (2002). Augmentation index as a measure of peripheral vascular disease state. Curr Opin Cardiol.

[11] Kelly R, Hayward C, Avolio A, O’Rourke M (1989). Noninvasive determination of age-related changes in the human arterial pulse. Circulation.

[12] Laurent S, Cockcroft J, Van Bortel L, Boutouyrie P, Giannattasio C, Hayoz D, Pannier B, Vlachopoulos C, Wilkinson I, Struijker-Boudier H (2006). Expert consensus document on arterial stiffness: methodological issues and clinical applications. Eur Heart J.

[13] Kenny D, Polson JW, Martin RP, Caputo M, Wilson DG, Cockcroft JR, Paton JFR, Wolf AR (2011). Relationship of aortic pulse wave velocity and baroreceptor reflex sensitivity to blood pressure control in patients with repaired coarctation of the aorta. Am Heart J.

[14] Iwasaki K, Kusachi S (2009). Coronary pressure measurement based decision making for percutaneous coronary intervention. Curr Cardiol Rev.

[15] Garcia L, Carrozza J (2007). Physiologic evaluation of translesion pressure gradients in peripheral arteries: comparison of pressure wire and catheter-derived measurements. J Interv Cardiol.

[16] Back LH (1994). Estimated mean flow resistance increase during coronary artery catheterization. J Biomech.

[17] Costard-Jäckle A, Fowler MB (1992). Influence of preoperative pulmonary artery pressure on mortality after heart transplantation: Testing of potential reversibility of pulmonary hypertension with nitroprusside is useful in defining a high risk group. J Am Coll Cardiol.

[18] Banner NR, Bonser RS, Clark AL, Clark S, Cowburn PJ, Gardner RS, Kalra PR, McDonagh T, Rogers CA, Swan L, Parameshwar J (2011). UK guidelines for referral and assessment of adults for heart transplantation. Heart.

[19] Cavendish JJ, Carter LI, Tsimikas S (2008). Recent advances in hemodynamics: noncoronary applications of a pressure sensor angioplasty guidewire. Catheter Cardiovasc Interv.

[20] Blows LJ, Redwood SR (2007). The pressure wire in practice. Heart.

[21] Bae J, Lerman A, Yang E, Rihal C (2006). Feasibility of a pressure wire and single arterial puncture for assessing aortic valve area in patients with aortic stenosis. J Invasive Cardiol.

[22] Gaddum NR, Schaeffter T, Bührer M, Rutten M, Smith L, Chowienczyk PJ, Beerbaum PBJ (2013). Beat-to-beat variation in pulse wave velocity during breathing maneuvers. Magn Reson Med.

[23] Peacock J, Jones T, Tock C, Lutz R (1998). The onset of turbulence in physiological pulsatile flow in a straight tube. Exp Fluids.

[24] Lee TS, Shi ZD (1999). Effects of Reynolds number on physiological-type pulsatile flows in a pipe with ring-type constrictions. Int J Numer Methods Fluids.

[25] Yao W, Schaeffter T, Seneviratne L, Althoefer K (2012). Developing a Magnetic Resonance-compatible catheter for cardiac catheterization. J Med Devices.

[26] Roeder R, Wolfe J, Lianakis N, Hinson T, Geddes LA, Obermiller J (1999). Compliance, elastic modulus, and burst-pressure of small-intestine submucosa (SIS) small-diameter vascular grafts. J Biomed Mater Res.

[27] Klee G, Ackerman E, Leonard A (1974). Computer detection of distortion in arterial pressure signals. IEEE Trans Biomed Eng.

[28] Nordsletten D, McCormick M, Kilner P, Hunter P, Kay D, Smith N (2011). Fluid–solid coupling for the investigation of diastolic and systolic human left ventricular function. Int J Numer Method Biomed Eng.

[29] de Vecchi A, Nordsletten D, Remme EW, Bellsham-Revell H, Greil G, Simpson JM, Razavi R, Smith NP (2012). Inflow typology and ventricular geometry determine efficiency of filling in the hypoplastic left heart. Ann Thorac Surg.

[30] Kannel WB (1981). Systolic Blood Pressure, Arterial Rigidity, and Risk of Stroke: The Framingham Study. JAMA J Am Med Assoc.

